# Association of Academic Stress, Physical Activity, Sedentary Behavior, and Diabetes Risk Among University Students

**DOI:** 10.3390/healthcare14131894

**Published:** 2026-06-29

**Authors:** Siti Nur Asiyah, Atik Qurrota A’yunin Al Isyrofi, Ayu Mei Wulandari, Ambarwati, Aini Nurul Fatimatuz Zahroh, Achmad Ilham Fanany Al Isyrofie

**Affiliations:** 1 Department of Public Health, Universitas Islam Negeri Sunan Ampel Surabaya, Surabaya 60237, Indonesia; ayu.wulandari@uinsa.ac.id (A.M.W.); ambarwati@uinsa.ac.id (A.); aini30nurul@gmail.com (A.N.F.Z.); 2 Department of Public Health, Universitas Nahdlatul Ulama Surabaya, Surabaya 60237, Indonesia; atikqurrotaa@unusa.ac.id; 3 Department of Biomedical Sciences, Universitas Airlangga, Surabaya 60115, Indonesia; achmadilhamfanany@gmail.com

**Keywords:** academic stress, diabetes risk, physical activity, sedentary behavior, university students

## Abstract

**Highlights:**

Academic stress was significantly associated with an increased risk of diabetes among university students. Sedentary behavior was associated with an increased metabolic risk. Moderate-to-vigorous physical activity was associated with a lower risk of diabetes. Exploratory factor analysis supported the construct validity of the academic stress instrument. Correlation and multivariable logistic regression analyses demonstrated consistent associations among academic stress, physical activity, sedentary behavior, and diabetes risk.

**What are the main findings?**

- Academic stress was significantly associated with an increased risk of diabetes among university students.
- Sedentary behavior was associated with an increased metabolic risk.
- Moderate-to-vigorous physical activity was associated with a lower risk of diabetes.
- Higher levels of academic stress and longer sitting duration were associated with greater diabetes risk.

**What are the implications of the main findings?**

- Universities should integrate stress management into health promotion programs.
- Physical activity promotion should be strengthened among university students.
- Strategies to reduce sedentary behavior may help prevent metabolic risk factors in young adults.
- Early psychosocial and lifestyle interventions may support long-term metabolic health.

**Abstract:**

Background: The increasing prevalence of diabetes mellitus and metabolic risk factors among young adults has become a major public health concern. University students are particularly vulnerable to unhealthy lifestyle changes, including sedentary behavior, insufficient physical activity, and academic stress, all of which may be associated with an elevated risk of metabolic disorders. Objective: This study aimed to examine the associations of academic stress, physical activity, and sedentary behavior with diabetes risk among university students. Methods: A cross-sectional analytical study was conducted among 264 university students recruited through an online survey. Academic stress was assessed using a six-item Likert-scale instrument, while diabetes risk was evaluated using a composite score derived from indicators adapted from the modified Finnish Diabetes Risk Score (modified FINDRISC). Statistical analyses included descriptive statistics, Cronbach’s alpha reliability testing, exploratory factor analysis (EFA), Spearman’s correlation analysis, and multivariable logistic regression. Results: The academic stress instrument demonstrated good internal consistency (Cronbach’s alpha = 0.85). Exploratory factor analysis supported the construct validity of the instrument, with all six items loading substantially on a common academic stress factor. Correlation analysis revealed that academic stress was positively associated with sedentary behavior and diabetes risk, whereas physical activity was negatively associated with diabetes risk. Multivariable logistic regression showed that academic stress was significantly associated with an increased risk of diabetes (adjusted odds ratio [aOR] = 1.18, 95% confidence interval [CI]: 1.02–1.36; *p* = 0.028). Moderate-to-vigorous physical activity was associated with a lower risk of diabetes (aOR = 0.74, 95% CI: 0.60–0.92; *p* = 0.011), while longer sitting duration was associated with an increased risk of diabetes. Conclusions: Academic stress, sedentary behavior, and physical activity were significantly associated with diabetes risk among university students. These findings highlight the importance of developing university-based health promotion programs that integrate stress management, physical activity promotion, and sedentary behavior reduction to support the prevention of metabolic risk factors in young adults.

## 1. Introduction

Diabetes mellitus has become one of the most significant global public health challenges due to its rapidly increasing prevalence, substantial morbidity, long-term complications, and considerable economic burden on healthcare systems [1]. According to the International Diabetes Federation (IDF), an estimated 537 million adults worldwide were living with diabetes in 2021, and this number is projected to increase to 643 million by 2030 and 783 million by 2045 [1]. Although diabetes has traditionally been associated with older populations, accumulating evidence suggests that metabolic risk factors are increasingly emerging among adolescents and young adults as a consequence of unhealthy lifestyle transitions, urbanization, physical inactivity, unhealthy dietary patterns, sleep disturbances, and psychological stress [2,3].

University students represent a particularly vulnerable population because the transition to higher education is often accompanied by substantial academic, social, psychological, and behavioral changes. They frequently experience increased academic workload, examination pressure, emotional exhaustion, irregular sleep patterns, reduced physical activity, prolonged screen exposure, and sedentary study habits [4]. These behavioral and psychosocial factors may be associated with the early development of metabolic disturbances, obesity, insulin resistance, and an elevated risk of type 2 diabetes mellitus later in life [5].

Academic stress has emerged as an important psychosocial determinant of health among university students. According to the biopsychosocial model and allostatic load theory, chronic psychological stress may disrupt physiological adaptive mechanisms and contribute to long-term metabolic dysregulation [6]. Persistent academic stress activates the hypothalamic–pituitary–adrenal (HPA) axis, resulting in prolonged cortisol secretion, autonomic nervous system dysregulation, oxidative stress, inflammatory cytokine production, and impaired glucose metabolism [7,8]. Elevated cortisol levels have been associated with insulin resistance, abdominal obesity, endothelial dysfunction, and metabolic syndrome [7].

In addition to psychological stress, sedentary behavior and insufficient physical activity have been recognized as important modifiable factors associated with cardiometabolic disease. The World Health Organization (WHO) has reported that physical inactivity is associated with an increased risk of obesity, cardiovascular disease, diabetes mellitus, and premature mortality [9]. Sedentary behavior, particularly prolonged sitting time and excessive screen exposure, may impair skeletal muscle glucose uptake, reduce energy expenditure, alter lipid metabolism, and promote chronic low-grade inflammation [10,11]. Emerging evidence suggests that sedentary behavior may be independently associated with diabetes risk even among individuals who meet recommended levels of physical activity [12].

The COVID-19 pandemic and the rapid digitalization of academic systems have further intensified sedentary lifestyles among university students. Online learning environments, increased reliance on electronic devices, and reduced opportunities for outdoor activities have substantially prolonged sitting time and decreased physical activity among young adults [13,14]. Several recent studies have reported worsening mental health, increased stress levels, unhealthy dietary behaviors, sleep disturbances, and reduced physical activity among university students during and after the pandemic period [15,16].

Previous studies have demonstrated associations among psychological stress, physical activity, sedentary behavior, and metabolic disorders across various populations, including young adults. However, research specifically examining the relationships among academic stress, physical activity, sedentary behavior, and diabetes risk among university students in Indonesia remains limited. Furthermore, few studies have integrated psychometric evaluation of academic stress instruments with multivariable statistical analyses to identify factors independently associated with an increased risk of diabetes.

Therefore, this study aimed to examine the associations among academic stress, physical activity, sedentary behavior, and diabetes risk among university students and to identify factors independently associated with increased diabetes risk.

## 2. Materials and Methods

### 2.1. Study Design and Setting

This cross-sectional analytical study was conducted to examine the associations among academic stress, physical activity, sedentary behavior, and diabetes risk among university students. Data were collected through an online survey distributed via digital communication platforms to undergraduate students in Surabaya, Indonesia. A cross-sectional design was chosen because it enables the simultaneous assessment of exposure variables and outcomes within a defined population during a specific study period.

### 2.2. Participants and Sampling

A total of 264 university students participated in this study. Participants were recruited through voluntary participation using an online questionnaire distributed via digital communication platforms. The inclusion criteria were (1) active undergraduate enrollment, (2) willingness to participate voluntarily, and (3) completion of all questionnaire items. A total of 264 complete responses were included in the final analysis. The online survey platform required completion of all questionnaire items before submission; therefore, no item-level missing data were observed in the final dataset.

**Figure 1:**
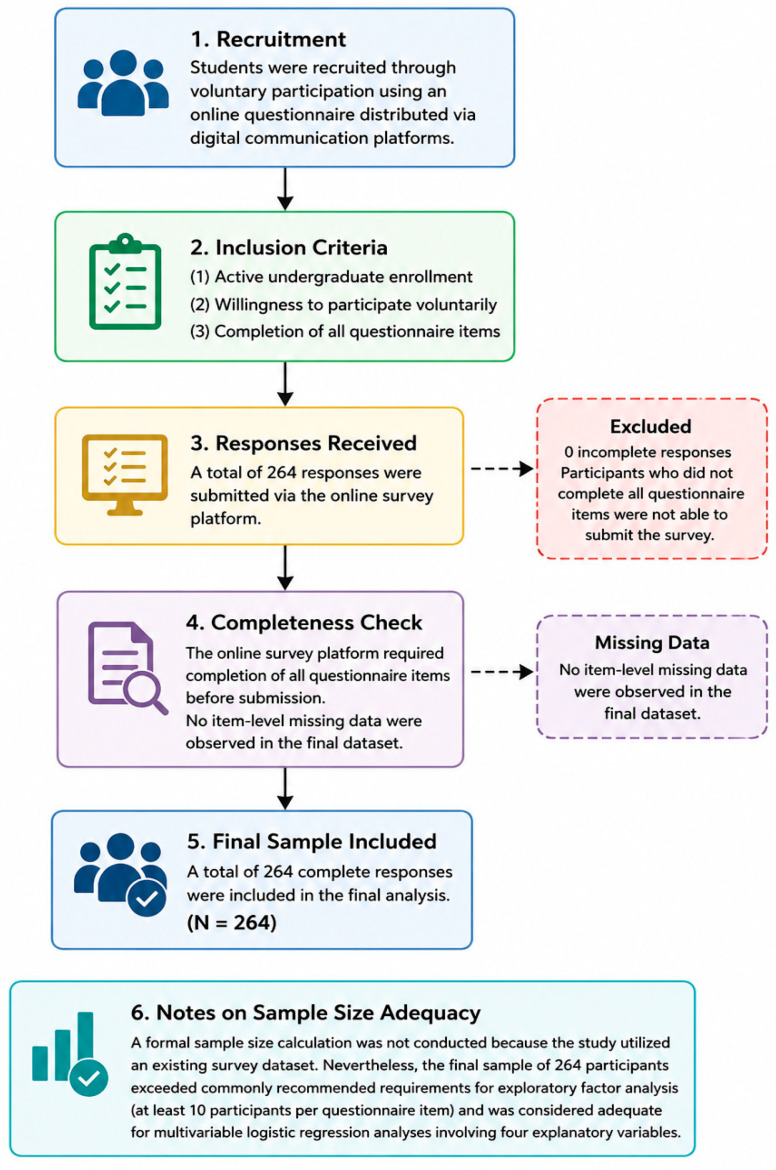
Flowchart of participant recruitment and inclusion in the study (N = 264). Dashed arrows indicate exclusion and data verification procedures.

A formal sample size calculation was not conducted because the study utilized an existing survey dataset. Nevertheless, the final sample of 264 participants exceeded commonly recommended requirements for exploratory factor analysis (at least 10 participants per questionnaire item) and was considered adequate for multivariable logistic regression analyses involving four explanatory variables. The participant recruitment and inclusion process is illustrated in Figure 1. 

### 2.3. Variables and Measurements

#### 2.3.1. Academic Stress

Academic stress was assessed using a six-item self-reported Likert-scale instrument designed to evaluate psychological burden related to academic activities. The instrument included items addressing feelings of being overwhelmed by academic tasks, difficulty managing academic stress, anxiety related to examinations and grades, inability to cope with academic demands, pressure arising from class schedules, and sleep disturbances caused by academic concerns. Responses were recorded on a five-point Likert scale ranging from 1 (strongly disagree) to 5 (strongly agree), with higher total scores indicating greater levels of academic stress. Detailed descriptions of the six questionnaire items are provided in Supplementary Table S1.

#### 2.3.2. Diabetes Risk Assessment

Diabetes risk was assessed using a modified composite score adapted from selected components of the Finnish Diabetes Risk Score (FINDRISC). The score included six variables available in the survey dataset: age, body mass index (BMI), family history of diabetes mellitus, physical activity, fruit and vegetable consumption, and history of elevated blood glucose. The components of the modified diabetes risk score are presented in Table 1.

Each risk factor was assigned a score of 0 (absence of risk) or 1 (presence of risk), resulting in a total score ranging from 0 to 6. Participants with a total score ≥ 2 were classified as having increased diabetes risk, whereas those with scores < 2 were classified as having lower diabetes risk. Because the study population consisted predominantly of young adults, the age component showed limited variability within the sample.

Because several original FINDRISC components (such as waist circumference and antihypertensive medication use) were unavailable, the modified score was intended solely as an epidemiological indicator of relative diabetes risk within the study population and not as a clinical diagnostic instrument.

#### 2.3.3. Physical Activity

Physical activity was assessed using self-reported questions adapted from the International Physical Activity Questionnaire (IPAQ) framework. Participants reported the frequency of walking, light-intensity physical activity, and moderate-to-vigorous physical activity performed during the previous seven days. The instrument was not intended to generate standard IPAQ metabolic equivalent (MET) scores but was used to describe participants’ physical activity patterns.

#### 2.3.4. Sedentary Behavior

Sedentary behavior was assessed using a self-reported sitting-time question adapted from the International Physical Activity Questionnaire (IPAQ) framework [17]. Participants reported their average daily sitting duration during routine activities, including academic work, studying, screen-based activities, transportation, and leisure time. A longer sitting duration was considered indicative of a more sedentary lifestyle.

### 2.4. Data Collection Procedure

Data were collected from February to March 2026 using a structured online questionnaire distributed through digital communication platforms. Before completing the survey, all participants received information regarding the study objectives, confidentiality procedures, voluntary participation, and the electronic informed consent process. Completion of the questionnaire took approximately 10–15 min. All collected data were anonymized before analysis to ensure participant confidentiality and privacy protection.

### 2.5. Statistical Analysis

Statistical analyses were conducted using IBM SPSS Statistics for Windows, Version 26.0 (IBM Corp., Armonk, NY, USA). Descriptive statistics were reported as frequencies, percentages, means, and standard deviations, as appropriate for the characteristics and distribution of the variables.

The internal consistency of the academic stress instrument was evaluated using Cronbach’s alpha, with values of 0.70 or higher considered indicative of acceptable reliability. Subsequently, exploratory factor analysis (EFA) was conducted to identify latent dimensions underlying the academic stress construct, with factor loadings of 0.40 or greater considered meaningful for interpretation.

Spearman’s rank correlation analysis was used to examine the relationships among academic stress, physical activity, sedentary behavior, and diabetes risk because several variables were ordinal and did not satisfy the assumption of normality.

To identify factors associated with diabetes risk, multivariable logistic regression analysis was performed. The regression model included academic stress, moderate-to-vigorous physical activity, sitting duration, and family history of diabetes as explanatory variables. These variables were selected based on conceptual considerations and previous evidence regarding metabolic risk factors among young adults. Analyses were conducted using complete-case data; therefore, no imputation procedures were applied for missing values. The results are presented as regression coefficients (β), standard errors (SEs), adjusted odds ratios (aORs), 95% confidence intervals (95% CIs), and *p*-values. A two-sided *p*-value of <0.05 was considered statistically significant.

### 2.6. Ethical Considerations

This study was approved by the Health Research Ethics Committee of STIKES Yayasan Rumah Sakit Dr. Soetomo, Surabaya, Indonesia (Ethics Approval No. KEPK/YRSDS/002/II/2026). The study protocol complied with ethical principles governing biomedical research involving human participants.

Participation was voluntary and anonymous. Electronic informed consent was obtained from all participants before completion of the questionnaire. Participants were informed about the study objectives, data confidentiality, the voluntary nature of participation, and their right to withdraw from the study at any time without penalty.

## 3. Results

### 3.1. Participant Characteristics

A total of 264 university students participated in this study, with a mean age of 19.75 ± 1.33 years. Most participants were female (90.2%), whereas males accounted for 9.8% of the study population. More than half of the participants had a body mass index (BMI) of ≥25 kg/m^2^ (59.1%), and approximately one-quarter (26.9%) reported a family history of diabetes mellitus.

Overall, 44.7% of participants reported inadequate fruit and vegetable consumption, while 39.0% had insufficient physical activity. Based on the modified composite diabetes risk score used in this study, 37.1% of participants were classified as having an increased diabetes risk, whereas 62.9% were categorized as having a lower diabetes risk. The mean academic stress score was 10.05 ± 4.30, with a median of 11 (range: 0–18), indicating variability in stress levels among participants. The baseline characteristics of the study participants are presented in Table 2.

### 3.2. Reliability Analysis

Reliability analysis demonstrated that the six-item academic stress scale achieved a Cronbach alpha coefficient of 0.85, indicating good internal consistency and satisfactory psychometric reliability. A Cronbach alpha value above 0.80 suggests that the questionnaire items consistently measured the underlying construct of academic stress. The reliability analysis results are presented in Table 3. 

### 3.3. Exploratory Factor Analysis

The suitability of the data for factor analysis was evaluated using the Kaiser–Meyer–Olkin (KMO) Measure of Sampling Adequacy and Bartlett’s Test of Sphericity. The KMO value was 0.874, indicating excellent sampling adequacy. Bartlett’s Test of Sphericity was statistically significant (χ^2^ = 595.49, df = 15, *p* < 0.001), confirming that the correlation matrix was appropriate for factor analysis.

Principal Component Analysis identified one dominant factor with an eigenvalue of 3.46, explaining 57.6% of the total variance. Based on the eigenvalue >1 criterion, a single factor was retained. All six items demonstrated substantial factor loadings ranging from 0.69 to 0.82, supporting the construct validity of the academic stress instrument.

Overall, the findings indicate that the instrument measures a common underlying construct of academic stress while capturing different manifestations of students’ academic experiences and psychological responses. The results are presented in Table 4.

As shown in Table 4, the KMO value of 0.874 indicated excellent sampling adequacy for factor analysis. Bartlett’s Test of Sphericity was statistically significant (χ^2^ = 595.49, df = 15, *p* < 0.001), confirming that the correlation matrix was suitable for factor extraction. Principal Component Analysis identified one dominant factor with an eigenvalue of 3.46, accounting for 57.6% of the total variance. Based on the eigenvalue > 1 criterion, a single factor was retained for interpretation. 

The factor loadings for each academic stress item are presented in Table 5.

All six items demonstrated substantial factor loadings ranging from 0.69 to 0.82, supporting the construct validity of the academic stress instrument. The findings suggest that the instrument measures a common underlying construct of academic stress while capturing different manifestations of students’ academic experiences and psychological responses. Because only one factor met the retention criterion (eigenvalue > 1), no rotation procedure was applied. 

### 3.4. Correlation Analysis

Spearman’s correlation analysis demonstrated a moderate positive correlation between academic stress and sedentary behavior (ρ = 0.41, *p* < 0.001), indicating that students experiencing higher levels of academic stress tended to spend more time in sedentary activities. Academic stress also showed a weak positive correlation with diabetes risk (ρ = 0.29, *p* < 0.001).

Conversely, physical activity was significantly and inversely correlated with both sedentary behavior and diabetes risk. Sedentary behavior showed a weak positive correlation with diabetes risk. The Spearman correlation analysis results are presented in Table 6.

### 3.5. Multivariable Logistic Regression Analysis

Multivariable logistic regression analysis was performed to identify factors independently associated with increased diabetes risk among university students.

Academic stress was significantly associated with diabetes risk (β = 0.166, SE = 0.073, aOR = 1.18, 95% CI: 1.02–1.36, *p* = 0.028). Specifically, each one-unit increase in the academic stress score was associated with an 18% increase in the odds of increased diabetes risk after adjustment for other variables included in the model.

Moderate-to-vigorous physical activity was significantly associated with lower diabetes risk (β = −0.301, SE = 0.109, aOR = 0.74, 95% CI: 0.60–0.92, *p* = 0.011), corresponding to an approximately 26% reduction in the odds of increased diabetes risk.

Longer sitting duration was also significantly associated with increased diabetes risk (β = 0.482, SE = 0.173, aOR = 1.62, 95% CI: 1.13–2.42, *p* = 0.019), indicating a 62% higher odds of increased diabetes risk.

Similarly, family history of diabetes was independently associated with increased diabetes risk (β = 0.747, SE = 0.283, aOR = 2.11, 95% CI: 1.24–3.76, *p* = 0.008). Participants with a family history of diabetes had approximately twice the odds of increased diabetes risk compared with those without such a history. The detailed results of the multivariable logistic regression analysis are presented in Table 7.

## 4. Discussion

This study found that academic stress and sedentary behavior were positively associated with increased diabetes risk, whereas moderate-to-vigorous physical activity was inversely associated with diabetes risk among university students. In addition, the academic stress instrument demonstrated satisfactory reliability and construct validity. These findings suggest that both psychosocial and behavioral factors may contribute to metabolic risk profiles in young adults. Multivariable logistic regression analysis showed that each one-unit increase in the academic stress score was associated with an 18% increase in the odds of diabetes risk (aOR = 1.18, 95% CI: 1.02–1.36). These findings are consistent with the biopsychosocial model and allostatic load theory, which propose that chronic psychological stress may disrupt physiological adaptive mechanisms and contribute to metabolic dysregulation [4,7]. University students are particularly susceptible to chronic stress because of academic demands, competitive learning environments, sleep disturbances, and various psychosocial pressures encountered during higher education [4].

The observed positive association between academic stress and diabetes risk may be explained by chronic activation of the hypothalamic–pituitary–adrenal (HPA) axis, leading to prolonged cortisol secretion and altered autonomic nervous system regulation [7]. Sustained elevations in cortisol have been associated with impaired glucose metabolism, insulin resistance, and inflammatory processes that contribute to the development of metabolic disorders [7,8].

In addition to psychological factors, this study found that sedentary behavior was significantly associated with increased diabetes risk. Multivariable regression analysis indicated that longer sitting duration was associated with a 62% increase in the odds of diabetes risk (aOR = 1.62, 95% CI: 1.13–2.42). This finding is consistent with previous studies reporting that prolonged sedentary behavior may impair glucose metabolism, reduce energy expenditure, and increase the risk of metabolic disorders [9,10,11]. Moreover, several studies have suggested that sedentary behavior remains associated with metabolic disease risk even among individuals who meet recommended levels of physical activity [10,11].

The relatively high prevalence of sedentary behavior observed in this study may reflect post-pandemic changes in learning patterns and the increasing use of digital technologies in education. Online learning, prolonged use of electronic devices, and reduced opportunities for outdoor activities have been reported to contribute to longer sitting duration among university students [13,14]. Previous studies have also shown that the COVID-19 pandemic was associated with increased psychological stress, reduced physical activity, and unfavorable changes in health-related behaviors among student populations [13,15].

The correlation and multivariable regression analyses yielded complementary findings. Academic stress was positively correlated with sedentary behavior and diabetes risk, whereas physical activity was negatively correlated with diabetes risk. These results suggest that psychological and lifestyle-related factors may interact in shaping metabolic risk among university students, highlighting the importance of addressing both domains in preventive strategies.

Conversely, moderate-to-vigorous physical activity was associated with a lower risk of diabetes. The regression analysis indicated that greater engagement in moderate-to-vigorous physical activity was associated with a 26% reduction in the odds of diabetes risk (aOR = 0.74, 95% CI: 0.60–0.92). This finding is supported by previous evidence demonstrating that regular physical activity improves insulin sensitivity, enhances glucose metabolism, reduces adiposity, and provides long-term benefits for cardiometabolic and mental health [16,17,18,19,20].

Because moderate-to-vigorous physical activity was included both as an explanatory variable in the regression model and as one of the components of the adapted composite diabetes risk indicator, the observed association should be interpreted with appropriate methodological caution. Therefore, these findings are intended to describe observational associations rather than to establish an independent predictive effect or a causal relationship.

In addition, family history of diabetes was significantly associated with increased diabetes risk among university students (aOR = 2.11, 95% CI: 1.24–3.76). This finding reinforces existing evidence that genetic predisposition and hereditary factors remain important determinants of metabolic disease risk, which may be further influenced by unhealthy lifestyle behaviors and chronic stress exposure [21].

The exploratory factor analysis supported the construct validity of the academic stress instrument. All six items demonstrated substantial factor loadings on a common academic stress factor, suggesting that the instrument adequately captures the underlying construct of academic stress among university students. Furthermore, the high Cronbach alpha coefficient indicates good internal consistency and supports the suitability of the instrument for use in this population.

From a practical perspective, the findings highlight the importance of developing university-based health promotion programs that integrate stress management, physical activity promotion, and sedentary behavior reduction. Comprehensive interventions targeting these domains may help reduce metabolic risk factors while supporting the overall health and well-being of university students [21].

Future research should employ longitudinal designs, include more diverse populations, and incorporate laboratory biomarkers to better elucidate the temporal relationships among academic stress, lifestyle behaviors, and metabolic disease risk in university students.

## 5. Limitations

This study has several limitations that should be considered when interpreting the findings. First, the cross-sectional design does not permit causal inferences regarding the relationships among academic stress, physical activity, sedentary behavior, and diabetes risk. Second, all data were collected through self-administered questionnaires, which may be subject to recall bias and self-reporting bias. Third, diabetes risk was assessed using a composite indicator adapted from the Finnish Diabetes Risk Score (modified FINDRISC) and was not confirmed through biochemical measurements such as fasting blood glucose or glycated hemoglobin (HbA1c). In addition, although multivariable logistic regression was used to identify factors associated with diabetes risk, more comprehensive model diagnostic procedures were not evaluated in this study; therefore, the findings should be interpreted with appropriate caution.

The primary objective of this study was to examine associations among variables using correlation analysis and multivariable logistic regression. Accordingly, the results should be interpreted as observational associations rather than evidence of individual predictive performance or causal relationships. Future studies should employ longitudinal designs, include more diverse populations, incorporate laboratory biomarkers, and report more comprehensive model diagnostics and measures of statistical precision to improve the validity and reproducibility of the findings.

Model fit statistics (e.g., Hosmer–Lemeshow goodness-of-fit test and pseudo-R^2^ measures) were not available because the analyses were reconstructed from archived datasets and therefore should be interpreted cautiously.

Furthermore, several variables included as explanatory factors in the regression analysis were also incorporated into the adapted composite diabetes risk indicator used as the study outcome. Consequently, the regression results should be interpreted as estimates of observational associations and should not be considered an independent prediction model or evidence of causality.

Finally, information on participants’ academic year and field of study was not collected in the survey and therefore could not be evaluated as potential factors associated with academic stress or diabetes risk.

## 6. Conclusions

Academic stress, sedentary behavior, and low levels of physical activity were significantly associated with increased diabetes risk based on the composite indicator used in this study. These findings underscore the importance of considering both psychological and lifestyle-related factors when addressing metabolic risk among university students.

The reliability analysis and exploratory factor analysis supported the psychometric properties of the academic stress instrument, while the correlation and multivariable logistic regression analyses provided consistent evidence of associations among academic stress, physical activity, sedentary behavior, and diabetes risk. Collectively, these findings support the potential value of university-based interventions that integrate stress management, physical activity promotion, and sedentary behavior reduction as part of broader health promotion strategies for young adults.

Overall, this study highlights the importance of developing comprehensive campus health promotion programs that address both psychological well-being and healthy lifestyle behaviors to support the early prevention of metabolic risk factors among university students.

## Figures and Tables

**Table 1:** Components of the modified diabetes risk score.

Variable	Score 0 (Absence of Risk)	Score 1 (Presence of Risk)
Age	Lower-risk category	Higher-risk category
Body mass index (BMI)	Normal BMI	Overweight/obesity
Family history of diabetes mellitus	No	Yes
Physical activity	Adequate	Inadequate
Fruit and vegetable consumption	Adequate	Inadequate
History of elevated blood glucose	No	Yes

**Table 2:** Baseline characteristics of the study participants (*n* = 264).

Characteristic	Value
Age (years), mean ± SD	19.75 ± 1.33
Male, *n* (%)	26 (9.8)
Female, *n* (%)	238 (90.2)
BMI ≥ 25 kg/m^2^, *n* (%)	156 (59.1)
Family history of diabetes mellitus, *n* (%)	71 (26.9)
Inadequate fruit and vegetable consumption, *n* (%)	118 (44.7)
Insufficient physical activity, *n* (%)	103 (39.0)
Increased diabetes risk (modified composite score), *n* (%)	98 (37.1)
Lower diabetes risk, *n* (%)	166 (62.9)
Academic stress score, mean ± SD	10.05 ± 4.30
Median (range)	11 (0–18)

**Table 3:** Reliability analysis of the academic stress instrument.

Instrument	Number of Items	Cronbach’s Alpha
Academic Stress Scale	6	0.85

**Table 4:** Suitability for Exploratory Factor Analysis.

Parameter	Value
KMO Measure of Sampling Adequacy	0.874
Bartlett’s Test χ^2^	595.49
Df	15
*p*-value	<0.001
Extraction Method	Principal Component Analysis
First Factor Eigenvalue	3.46
Variance Explained (%)	57.6

**Table 5:** Exploratory Factor Analysis Results.

Item	Factor Loading
Feeling overwhelmed by academic tasks	0.79
Difficulty managing stress	0.82
Anxiety related to grades	0.74
Inability to cope with academic demands	0.76
Pressure from class schedules	0.69
Sleep difficulties	0.81

**Table 6:** Spearman’s correlation analysis.

Variables	ρ (rho)	*p*-Value	Interpretation
Academic stress vs. sedentary behaviour	0.41	<0.001	Moderate positive correlation
Academic stress vs. diabetes risk	0.29	<0.001	Weak positive correlation
Physical activity vs. diabetes risk	−0.39	<0.001	Weak-to-moderate negative correlation
Sedentary behavior vs. diabetes risk	0.36	<0.001	Weak positive correlation

**Table 7:** Multivariable logistic regression results for diabetes risk.

Variable	β	SE	aOR	95% CI	*p*-Value
Academic stress	0.166	0.073	1.18	1.02–1.36	0.028
Moderate-to-vigorous physical activity	−0.301	0.109	0.74	0.60–0.92	0.011
Sitting duration	0.482	0.173	1.62	1.13–2.42	0.019
Family history of diabetes	0.747	0.283	2.11	1.24–3.76	0.008

## Data Availability

The data presented in this study are available from the corresponding author upon reasonable request. The data are not publicly available because of ethical and privacy considerations.

## Supplementary Materials

The following supporting information can be downloaded at https://www.mdpi.com/article/10.3390/healthcare14131894/s1, Table S1. Items of the Academic Stress Instrument.
