# Supplementary material for: Association of Academic Stress, Physical Activity, Sedentary Behavior, and Diabetes Risk Among University Students
Source: Healthcare (Basel). 2026 Jun 29;14(13):1894. doi: 10.3390/healthcare14131894 (PMC13361608; doi:10.3390/healthcare14131894)
Supplement: Supplementary file 1 [file healthcare-14-01894-s001.zip › healthcare-4347153-supplementary.pdf]

Supplementary Table S1. Items of the Academic Stress Instrument

No.	Item
1	I feel overwhelmed by the academic tasks that I am required to complete.
2	I have difficulty managing stress related to academic activities.
3	I feel anxious about my grades or upcoming examinations.
4	I feel unable to cope with academic demands.
5	I feel pressured by my busy class schedule or academic workload.
6	I experience difficulty sleeping because I worry about academic tasks or problems.

Note: All items were assessed using a five-point Likert scale (1 = strongly disagree to 5 = strongly agree), with higher scores indicating greater levels of academic stress.
